# Mycophenolate mofetil‐induced peripheral neuropathy in the treatment of membranous glomerulonephropathy: A case report

**DOI:** 10.1002/ccr3.5161

**Published:** 2021-12-13

**Authors:** Minoo Moghimi, Zahra Nekoukar, Farhad Gholami

**Affiliations:** ^1^ Department of Clinical Pharmacy Mazandaran University of Medical Sciences Sari Iran; ^2^ Department of Internal Medicine Faculty of Medicine Mazandaran University of Medical Sciences Mazandaran Iran

**Keywords:** membranous glomerulonephropathy, mycophenolate mofetil, peripheral neuropathy

## Abstract

Mycophenolate mofetil (MMF) as an immunosuppressive agent is widely used in the management of Membranous Glomerulonephropathy (MGN). In this report, we described a 66‐year‐old male MGN case treated with MMF and revealed acquired sensory‐motor axonal polyneuropathy, which is rare and has not been reported before.

## Graphical abstract

Although mycophenolate mofetil is effective in the management of some immune‐mediated neuromuscular disorders, it could inversely cause peripheral neuropathy as a rare side effect, which is hopefully reversible with drug discontinuation.

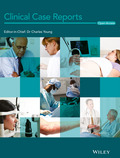

## 1 INTRODUCTION

Membranous glomerulonephropathy (MGN) is an autoimmune progressive disorder in which deposition of immune complexes occurred in the subepithelial space. It is commonly associated with nephrotic syndrome and can cause end‐stage renal disease (ESRD) in long term. It is usually seen as an idiopathic form (primary MGN), but the etiology can be diagnosable (secondary MGN). MGN typically presented with nephrotic syndrome (80%) or persisting non‐nephrotic proteinuria (20%). Patients with asymptomatic non‐nephrotic proteinuria usually responsed to conservative therapy.^1^ Indication of immunosuppressive treatment in idiopathic MGN include increased creatinine level at presentation, progressive disease, severe symptomatic nephrotic syndrome, thromboembolism, persistent nephrotic syndrome, male sex, and age older than 50 years, increased IgG excretion, HLA‐DR3 +/B8 +, white race, and elevation of urinary excretion of complement activation products, and tubulointerstitial changes or focal sclerosis.^2^

Immunosuppressive drugs that are commonly used to treat MGN include steroids, cyclophosphamide, chlorambucil, mycophenolate mofetil (MMF), cyclosporine, tacrolimus, and rituximab, an anti‐CD20 antibody directed at B cells.^1^

According to “The 2012 Kidney Disease Improving Global Outcomes” guidelines, a 6‐month course of alternating monthly cycle therapy with intravenous and oral glucocorticoids in combination with oral alkylating agents can reduce the progression of disease.^3^ Remission and reduction in nephrotic‐range proteinuria are eventuated with cyclosporine and tacrolimus, while relapses are frequent after discontinuation of the drug.^4^ Just like the combination mentioned above, administration of MMF with steroids obtains remission in 70% of patients.^5^ MMF may consider in patients with contraindication for alkylating agents, and those are at risk of renal damage associated with calcineurin inhibitors.^2^

### 1.1 Pharmacology and indications of MMF

MMF is an ester prodrug converted to its active metabolite Mycophenolic acid with immunosuppressive properties. MMF blocks inosine monophosphate dehydrogenase, a key enzyme for guanosine nucleotides synthesis leading to inhibition of T and B lymphocyte proliferation.^6^ It used for a variety of collagen‐vascular diseases like systemic lupus erythematosus.^7^

Also, MMF has been used in neuropathic conditions including polymyositis, chronic inflammatory demyelinating polyradiculoneuropathy, and multifocal motor neuropathy. Primarily, it is used as adjunctive therapy for steroid‐sparing effect or reducing the administration frequency of IVIG.^8^ It has an acceptable safety profile and also can be used as monotherapy in some patients.

### 1.2 Adverse effects of MMF

MMF affects multiple body organs, including cardiovascular, endocrine, gastrointestinal, genitourinary, respiratory, neuromuscular, hematologic, and central nervous systems, skin, liver, and kidney. Considering its immunosuppressive effects, the patient becomes susceptible to bacterial, viral (cytomegalovirus), and fungal infections.^9^

## 2 CASE PRESENTATION

A 66‐year‐old Iranian male patient was referred to the emergency room because of weakness, imbalance, lethargy, and tremor from 10 days ago. His past medical history revealed a 3‐month MGN diagnosis. His medical history consisted prednisolone 100 mg/day, MMF 1500 mg/day, spironolactone 100 mg/day, aspirin 80 mg/day, chlordiazepoxide 5 mg/day, and tamsulosin 0.8 mg/day.

Physical examination showed a lethargic man with an oral temperature of 37°C, blood pressure 100/60 mm Hg, pulse rate 74 beats/min, respiratory rate 18 breaths/minute, and oxygen saturation of 100% on room air. The patient had lost a great deal of sensation of touch, pain, vibration, feeling of warmth, and a sense of joint position symmetrically in the upper and particularly lower limbs. Also, progressive weakness and impaired motor function were in limbs, from distal to the proximal, predominantly in the lower extremities. A significant reduction in tendon reflexes in the limbs was noted. The legs twitched while walking, making the patient prone to fall. Symptoms of autonomic involvement, including hypotension, diaphoresis, and eye dryness, were not reported. However, the patient revealed bladder and bowel dysfunction. Electromyogram (EMG) demonstrated reduced proximal force in upper and lower limbs [Table 1], and muscle enzymes were in the normal range. No abnormality on abdominal and pelvic ultrasonography was detected. Neurology, infectious, cardiology, gastrointestinal, and hematology assessments did not show any problems related to the patient's symptoms. Other causes of neurotoxicity also ruled out. Regarding the clinical pharmacist's recommendation, the patient's drug history was evaluated for contributing to these manifestations. Accordingly, prednisolone tapered down to a maintenance dose of 60 mg/day and then 45 mg/day concomitantly starting vitamin B1 300 mg/day and L‐Carnitine 500 mg/day, which were not effective. Subsequently, after the discontinuation of MMF and substitution of cyclosporine, the patient was able to stand on his feet, walk with a cane, and perform his self‐care activities such as defecation and urination after a week. EMG results also confirmed these observed improvements. After one, three, six, and twelve months of follow‐up, the primary disease, MGN, was well controlled and he had no problem with walking. The capillary serum zone electrophoresis test also revealed an ameliorative trend at the 3rd and the 6th months [Table 2]. Written informed consent was obtained from the patient for the publication of this report.

1. Compound muscle action potentials and sensory nerve action potentials are unobtainable or low amplitude.
2. Neurogenic defects are seen in some sampled muscle.

**TABLE 1:** EMG findings. Clinical remarks: Weakness and paresthesia in all limbs

A: NCS					
Motor nerves	Amplitude (mv)	Distal latency (ms)	NCV (m/s, >48)	F‐Wave latency (ms)	H‐Reflex (ms)
Rt. Tibial	Unobtain	–	–	–	Unobtain
Rt. DPN	Unobtain	–	–	–	–
Lt. Tibial	Unobtain	–	–	–	Unobtain
Lt. DPN	Unobtain	–	–	–	–
Rt. Median	2.1	5.8	–	–	–
Rt. Ulnar	1.4	6.0	–	–	–
Lt. Median	1.7	5.9	–	–	–
Lt. Ulnar	0.8	5.4	–	–	–

B: Needle EMG									
Muscle	IA	Spontaneous activity				MUAP			
		Fib.	PSW	Fasc.	Others	Amplitude	Duration	Polyphasic	Pattern
Lt.Tib.Ant	Inc	2+	2+	0	0	Inc	Inc	Inc	Single
Lt. Per.long	Inc	2+	2+	0	0	Inc	Inc	Inc	Single
Lt.GCS	Inc	2+	2+	0	0	Inc	Inc	Inc	Single
Lt.VMO	Inc	0	0	0	0	Inc	Inc	Inc	Discrete
Lt.Add.Mag	Inc	0	0	0	0	Inc	Inc	Inc	Discrete
Rt.Tib.Ant	Inc	2+	2+	0	0	Inc	Inc	Inc	Single
Rt.Per.long	Inc	2+	2+	0	0	Inc	Inc	Inc	Single
Lt.FCR	Inc	0	0	0	0	Inc	Inc	Inc	Discrete
Lt.EDC	Inc	0	0	0	0	Inc	Inc	Inc	Discrete
Lt.Biceps	Inc	0	0	0	0	Inc	Inc	Inc	Discrete
Rt.EDC	Inc	0	0	0	0	Inc	Inc	Inc	Discrete
Rt.FCR	Inc	0	0	0	0	Inc	Inc	Inc	Discrete

Abbreviations: Fasc, fasciculation; Fib, fibrillation; H‐Reflex, Hoffmann Reflex; IA, insertion activity; MUAP, motor unit action potential; NCS, nerve conduction studies; NCV, nerve conduction velocity; PSW, positive sharp waves.

**TABLE 2:** Capillary serum zone electrophoresis

Date	Fraction	%	Ref.%	g/dl	Ref. g/dl
Baseline	Albumin	32.2 L	55.8–66.1	1.58	4.02–4.76
	Alpha 1	5.1 H	2.9–4.9	0.25	0.21–0.35
	Alpha 2	29.0 H	7.1–11.8	1.42	0.51–0.85
	Beta 1	4.8	4.7–7.2	0.24	0.34–0.52
	Beta 2	10.9 H	3.2–6.5	0.53	0.23–0.47
	Gamma	18.0	11.1–18.8	0.88	0.80–1.35
Comments	T.P.: 4.9			A/G ‐ Ratio: 0.47	
First follow‐up (3rd month)	Albumin	53.9 L	55.8–66.1	3.18	4.02–4.76
	Alpha 1	5.8 H	2.9–4.9	0.34	0.21–0.35
	Alpha 2	21.3 H	7.1–11.8	1.26	0.51–0.85
	Beta 1	5.9	4.7–7.2	0.35	0.34–0.52
	Beta 2	4.3	3.2–6.5	0.25	0.23–0.47
	Gamma	8.8 L	11.1–18.8	0.52	0.80–1.35
Comments	T.P.: 5.9			A/G ‐ Ratio: 1.17	
Second follow‐up (6th month)	Albumin	48.2 L	55.8–66.1	3.0	4.02–4.76
	Alpha 1	6.8 H	2.9–4.9	0.35	0.21–0.35
	Alpha 2	18.9 H	7.1–11.8	1.2	0.51–0.85
	Beta 1	7.1	4.7–7.2	0.4	0.34–0.52
	Beta 2	5.0	3.2–6.5	0.3	0.23–0.47
	Gamma	14.0	11.1–18.8	0.9	0.80–1.35
Comments	T.P.: 6.3			A/G ‐ Ratio: 0.93	

Abbreviations: A/G, albumin to glubolin; H, high; L, low; T.P., total protein.

### 2.1 Impression

These finding are compatible with neurogenic progressive process. Acquired sensory‐motor axonal polyneuropathy would be the main impression.

## 3 DISCUSSION

Peripheral neuropathy results from the dorsal route ganglia damage, which is triggered by different etiologies. Medications are one of the common causes of peripheral neuropathy named as drug‐induced peripheral neuropathy (ِDIPN). Covalent modification, organelle damage, intracellular inflammatory signaling, axonal transport defects, and channelopathies are the proposed mechanisms of DIPN.^10^ DIPN involves sensory nerves resulting in paresthesia and is reversible if diagnosed before significant axonal damage. After weeks to months of exposure, the signs and symptoms of peripheral neuropathy start in a dose‐dependent manner.^11^ Some antimicrobial, psychotropic, anticonvulsants, immunosuppressive, chemotherapeutic, and cardiovascular agents are known causes of DIPN. Risk factors contributed to DIPN incidence are diabetes, metabolic diseases, preexisting neuropathy, and genetic predisposition.^12^

Despite MMF reveals beneficial effects in immune‐mediated neuromuscular disorders, it inversely caused progressive neuropathy in our patient, which has not been reported before.

Most DIPNs cause mild sensory peripheral neuropathy, while our patient also suffered from even significant neurogenic and motor (defecation, urination, and walking) impairments. Hopefully, DIPN usually needs no specific intervention and improves only with dose reduction or drug discontinuation. In this case, following no improvement in the clinical status by discontinuing the suspicious drugs, the decision was made on MMF cessation, leading to neuropathy improvement.

## 4 CONCLUSION

Although MMF is one of the effective drugs in the management of immune‐mediated neuromuscular disorders, it could inversely cause peripheral neuropathy as a rare side effect. Hopefully, it can be recovered completely by dose reduction or cessation of the treatment.

## CONFLICT OF INTEREST

The authors confirm that this article content has no conflict of interest.

## AUTHOR CONTRIBUTIONS

All authors contributed to the preparation of this manuscript and have read and approved the final manuscript. FG was the patient's nephrologist who managed the peripheral neuropathy and was responsible for following up with the patient. MM involved in the interpretation and collecting of data, drafting the first version of the manuscript, and editing of it. ZN involved in drafting the first version of the manuscript and editing it.

## ETHICAL APPROVAL

This study was conducted according to the declaration of Helsinki principles. Also, CARE guidelines and methodology were followed in this study.

## CONSENT

Informed consent for the publication of this case report was obtained from the patient.

## Data Availability

The data are available with the correspondence author and can be achieved on request.
